# Growth hormone supplementation during ovarian stimulation in women with advanced maternal age undergoing preimplantation genetic testing for Aneuploidy

**DOI:** 10.1186/s13048-023-01279-y

**Published:** 2023-10-19

**Authors:** Yilun Sui, Min Xiao, Jing Fu, Lu Li, Yining Xu, Caixia Lei, Xiaoxi Sun

**Affiliations:** Shanghai Ji Ai Genetics and IVF Institute, Obstetrics and Gynecology Hospital, Fudan University, No. 352 Dalin Road, Huangpu District, Shanghai, People’s Republic of China

**Keywords:** Growth hormone, Advanced maternal age, Preimplantation genetic testing for aneuploidy, Euploidy, In vitro fertilization

## Abstract

**Background:**

Studies have shown that supplementation with recombinant human GH (rh-GH) during ovarian stimulation (OS) may improve the ovarian response and clinical outcomes of IVF. However, it remains unclear whether GH is associated with the ploidy status of embryos, and therefore, is unable to explain the underlying reason for the effect of GH on IVF outcomes. This study aimed to investigate whether GH supplementation in women with advanced maternal age (AMA) during OS is related to an increased probability of obtaining euploid blastocysts.

**Methods:**

This was a single center retrospective cohort study. The data of all women aged 38–46 years who underwent their first preimplantation genetic testing for aneuploidy (PGT-A) cycle between January 2021 and June 2022 were reviewed. Patients in the GH group received 4 IU/day subcutaneous GH supplementation from the beginning of OS to the trigger day, and patients in the control group did not. A total of 140 patients in the GH group and 272 patients in the control group were included after 1:2 propensity score matching.

**Results:**

The baseline and cycle characteristics between the two groups were similar. The proportion of cycles which obtained euploid blastocysts was significantly higher in the GH group than that in the control group (41.43% vs. 27.21%, P = 0.00). The GH group had a significantly higher euploid blastocyst rate per cohort (32.47% vs. 21.34%, P = 0.00) and mean euploid blastocyst rate per cycle (per biopsy cycle 0.35 ± 0.40 vs. 0.21 ± 0.33, P = 0.00; per OS cycle 0.27 ± 0.38 vs. 0.16 ± 0.30, P = 0.02). However, the benefit of GH was more significant in patients aged 38–40 years, but not significant in patients aged 41–46 years. Pregnancy outcomes were similar between the two groups after embryo transfer.

**Conclusions:**

GH supplementation during OS is associated with a significantly increased probability of obtaining euploid blastocysts in women aged 38–40 years, but this benefit is not significant in women aged 41–46 years. Our results explained the underlying reason for the effect of GH on IVF outcomes in existing studies, and might be helpful for AMA patients undergoing PGT-A cycles to obtain a better outcome meanwhile to avoid over-treatment.

**Trial registration:**

NCT05574894, www.clinicaltrials.gov.

## Background

With the postponement of women’s marriage and childbearing in recent decades, the number of women with advanced maternal age (AMA) seeking in vitro fertilization (IVF) has increased significantly. However, owing to the reduction of euploid embryos in women with AMA, they experience a higher prevalence of IVF failure, pregnancy loss, and congenital disabilities in offspring compared to younger women [1]. Studies have shown that the proportion of euploid blastocysts in women aged 27–35 years remained constant at approximately 55% and decreased rapidly to approximately 35% at the age of 38 years and less than 20% at the age of 43 years [2]. Preimplantation genetic testing for aneuploidy (PGT-A) can improve ongoing pregnancy and live birth outcomes per embryo transfer [3] and is considered beneficial for women with AMA undergoing IVF [4–6]. However, PGT-A is a screening technique that cannot fundamentally increase the number or proportion of euploid embryos in patients with AMA [7]. Therefore, obtaining more euploid embryos in the process of ovarian stimulation (OS) during IVF has become a challenge for doctors and patients with AMA [8].

The growth hormone (GH) is a polypeptide hormone secreted by the anterior pituitary gland, which plays a role in promoting cell division and growth by directly binding to receptors on target cells or stimulating the liver to secrete insulin-like growth factor (IGF) [9]. In 2015, Weall et al. demonstrated for the first time the presence of the GH receptor (GHR) in human oocytes and revealed that GHR expression in oocytes from women ≥ 35 years of age was significantly lower than that of younger women < 35 years [10]. The study group showed that GH supplementation could upregulate GHRs in oocytes, increase functional mitochondria, improve oocyte quality, and increase the number of high-quality embryos [10]. In addition, GH supplementation can reduce oxidative stress-related mitochondrial damage to improve the quality of oocytes [11] and reverse FOS and JUN family proteins-related apoptosis to increase the number of oocytes [12]. Thus, GH has become a prospective adjuvant in IVF, especially in women older than 35 years.

In recent years, studies have shown that supplementation with recombinant human GH (rh-GH) during OS may improve the ovarian response and clinical outcomes of IVF. A study of preovulatory ovarian follicles and follicular fluid showed that GH could upregulate the expression of follicular-stimulating hormone (FSH) and luteinizing hormone (LH) receptors in granulosa cells in women aged 39–45 years, thereby improving ovarian response and pregnancy outcomes in this population [13]. A recent meta-analysis evaluated the effect of GH in poor responders undergoing IVF and showed that GH supplementation reduced the dosage of gonadotropins for OS, increased the number of retrieved oocytes and transferrable embryos, and improved the clinical pregnancy rate [14]. However, as most of the existing research regarding GH in IVF was carried out in poor responders, there is a lack of studies focusing on women with AMA. Furthermore, the previous studies were conducted in the setting of untested embryos, thus it remains unclear whether the biological mechanisms underlying the observed effect of GH on the clinical pregnancy and live birth outcomes were embryo-mediated or endometrium-mediated.

Obtaining euploid embryos is a prerequisite for healthy live births and is considered the primary goal for OS in women with AMA. It is important to evaluate the effect of GH supplementation on the ploidy status of blastocysts in this population and therefore explain the underlying reason for the effect of GH on IVF outcomes. Thus, the present study aimed to compare the euploidy status of blastocysts obtained from patients with AMA with or without GH supplementation during OS.

## Methods

### Study design and population

This cohort study retrospectively collected the data of all women aged 38–46 years who underwent their first OS cycle scheduled for PGT-A in Shanghai JiAi Genetics and IVF Institute from January 2021 to June 2022 in the institutional electronic database. Each participant was required to have a body mass index (BMI) in the normal range (18.50–24.0 kg/m^2^), an antagonist protocol for OS, and a normal semen analysis for the male partner. Patients who had undergone previous failed IVF cycles before their PGT-A cycle were not excluded from the study. For patients with multiple PGT-A cycles during the study period, only the data from the first OS cycle were included. The exclusion criteria were as follows: (1) patients with endometriosis, untreated hydrosalpinx, or uterine abnormalities (such as adenomyosis, submucosal myoma, non-submucous myoma > 4 cm, and with compressed endometrium or uterine cavity lesions); (2) history of any other endocrine disorder, such as polycystic ovary syndrome or abnormal thyroid stimulating hormone, free T3, or free T4 levels; (3) history of autoimmune diseases or diagnosed thrombophilia; (4) patients indicated for preimplantation genetic testing for structural rearrangement or preimplantation genetic testing for monogenic disorder, such as parental abnormal karyotype or being diagnosed with monogenic disease; (5) history of smoking, radio- or chemotherapy; (6) history of GH supplementation in previous OS cycles or transfer cycles; (7) any other adjuvant drugs used during OS, such as DHEA, coenzyme Q10, recombinant human LH (rh-LH), proprietary Chinese medicine, or traditional Chinese medicine.

The women included were offered GH or not during OS at the discretion of the attending physicians or subject to the wishes of the couple after extensive counseling. Patients in the GH group received 4 IU/day subcutaneous rh-GH (Saizen, Changchun GeneScience, Changchun, China) from the beginning of OS to the trigger day, which was a routine dosage of GH supplementation in our IVF center and was also suggested by other studies [15–17]. Patients in the control group did not receive any GH supplementation. To attain a convincing result, these two groups were compared with a matched baseline and stimulation characteristics using propensity score matching (PSM) to avoid selection bias and adjust for confounding factors related to aneuploidy.

The study protocol was approved by the Research Ethics Committee of the Shanghai JiAi Genetics and IVF Institute (Approval Number: JIAI E2022-14, Study ID: JIAI E2022-022, NCT05574894, www.clinicaltrials.gov). All participants provided written informed consent.

### IVF-ET procedures and PGT-A

OS, oocyte retrieval, fertilization, blastocyst culture, endometrial preparation, embryo transfer, and luteal phase support were performed according to standard protocols in our IVF center, as previously described in detail [18, 19].

Briefly, a flexible antagonist protocol for controlled ovarian hyperstimulation was used for each participant, with recombinant human FSH (Gonal-f; Merck Serono, Geneva, Switzerland) or human menopausal gonadotropin (HMG, Livzon, Zhuhai, China) initiated on the 2nd or 3rd day of the menstrual cycle at a starting dose of 150–300 IU/day, adjusted for age, BMI, antral follicle count (AFC), FSH, and anti-Mullerian hormone (AMH) levels. Gonadotropin-releasing hormone antagonist (Cetrotide; Merck Serono) was administered at a dose of 0.25 mg/day when the dominant follicle reached 14 mm in size or the serum E2 level reached 350 pg/ml. This treatment continued until the leading follicle reached 18 mm or two follicles reached 16 mm in size. Subsequently, a dose of 5,000–10,000 IU of human chorionic gonadotropin (Livzon, Zhuhai, China) was administered as a trigger, and oocytes were retrieved 36 h later.

Intracytoplasmic sperm injection and blastocyst culture were performed for all participants following IVF laboratory guidelines, and next-generation sequencing-based PGT-A was administered to all blastocysts obtained using a NextSeq CN500 sequencer (Illumina, Inc. San Diego, CA, USA) according to the manufacturer’s instructions [19]. Mosaicism calls were made when 20–80% of the biopsied cells were aneuploid. All participants in the two groups with euploid embryos underwent single euploid blastocyst transfers from the second menstrual cycle after OS to within one year after OS. For patients with extra euploid embryos who experienced transfer failure, another single embryo transfer cycle will be performed until all euploid blastocysts acquired in the cycle were transferred. Luteal phase support was continued until 11 weeks of gestation if pregnancy was achieved with oral dydrogesterone (Duphaston, Abbott Biologicals, Netherlands; 20 mg per day) and vaginal progesterone gel (Crinone, Merck Serono; 90 mg per day).

### Study outcomes

The primary goal of a scheduled PGT-A cycle is to obtain euploid blastocysts for transfer. Therefore, the primary outcome was the proportion of cycles which obtained euploid blastocysts, calculated as the number of cycles with ≥ 1 euploid blastocyst divided by the total number of OS cycles in a cohort. Secondary outcomes included euploid blastocyst rate per cohort (total number of euploid blastocysts in a cohort/total number of biopsied embryos in the same cohort) and euploid blastocyst rate per cycle (number of euploid blastocysts obtained in a cycle/number of blastocysts obtained in the same OS cycle). The mean euploid blastocysts per cycle were calculated separately considering the OS cycle and biopsy cycle. A biopsy cycle was defined as the OS cycle with blastocysts for biopsy and genetic testing. If no blastocysts or euploid blastocysts were obtained after OS, the euploidy rate of this cycle was zero. Additional outcomes of interest included embryo implantation, clinical pregnancy, and ongoing pregnancy. Embryo implantation was defined as positive serum β-HCG levels 14 days after embryo transfer. Clinical pregnancy was defined as the visualization of the gestational sac on ultrasonography. Ongoing pregnancy was confirmed if a pulsating fetal pole was present at 12 weeks of gestation. Live birth was deliveries ≥ 22 weeks gestation with heartbeat and breath. All transfer outcomes were followed up until June 2023.

### Statistical analysis

PSM was used to identify the patients with or without GH supplement who were most similar in baseline and stimulation characteristics and to adjust for confounders related to aneuploidy. The variables in the PSM included indications for PGT-A, age, BMI, number of previous OS cycles, basal estradiol (E2), FSH, AMH, and AFC levels, duration of stimulation, and total dosage of gonadotropins. The PSM was carried out using a caliper width of 0.2 of the standard deviation (SD) of the logit of the propensity score and 1:2 ratio by nearest neighbor matching. Women who were not matched were excluded from further analyses.

According to the data from our IVF center, approximately 25% of patients with AMA can obtain ≥ 1 euploid blastocyst in a PGT-A cycle (unpublished data). As this proportion is supposed to be 40% in patients with AMA after GH supplement according to a previous study [20], a minimum of 117 patients in the GH group and 234 patients in the control group were needed to detect such a difference with 80% statistical power and a two-sided 0.05 level of significance, as calculated by PASS2021 software.

Values are presented as average ± SD for continuous data and compared using Student’s t-test or Mann–Whitney U test. Categorical variables were expressed as frequency and percentage, and between-group differences were assessed using Pearson’s chi-square test or Fisher’s exact test as appropriate. Subgroup analyses were performed stratified by age groups [21]. Considering whether a patient acquired euploid blastocysts in the OS cycle as the binary outcome, we used multivariate logistic regression to explore the relationships between GH supplementation and acquisition of euploid embryos while adjusting for age, AFC, AMH, and other possible confounders that may affect euploidy status determined by their clinical and statistical significance. Data were analyzed using Statistical Package for the Social Sciences (SPSS) software (version 26.0, SPSS, Inc., Chicago, IL, USA). The statistical significance level for all tests was set at P < 0.05.

## Results

### Baseline and cycle characteristics of patients in the GH and control groups before and after PSM

**Fig. 1:**
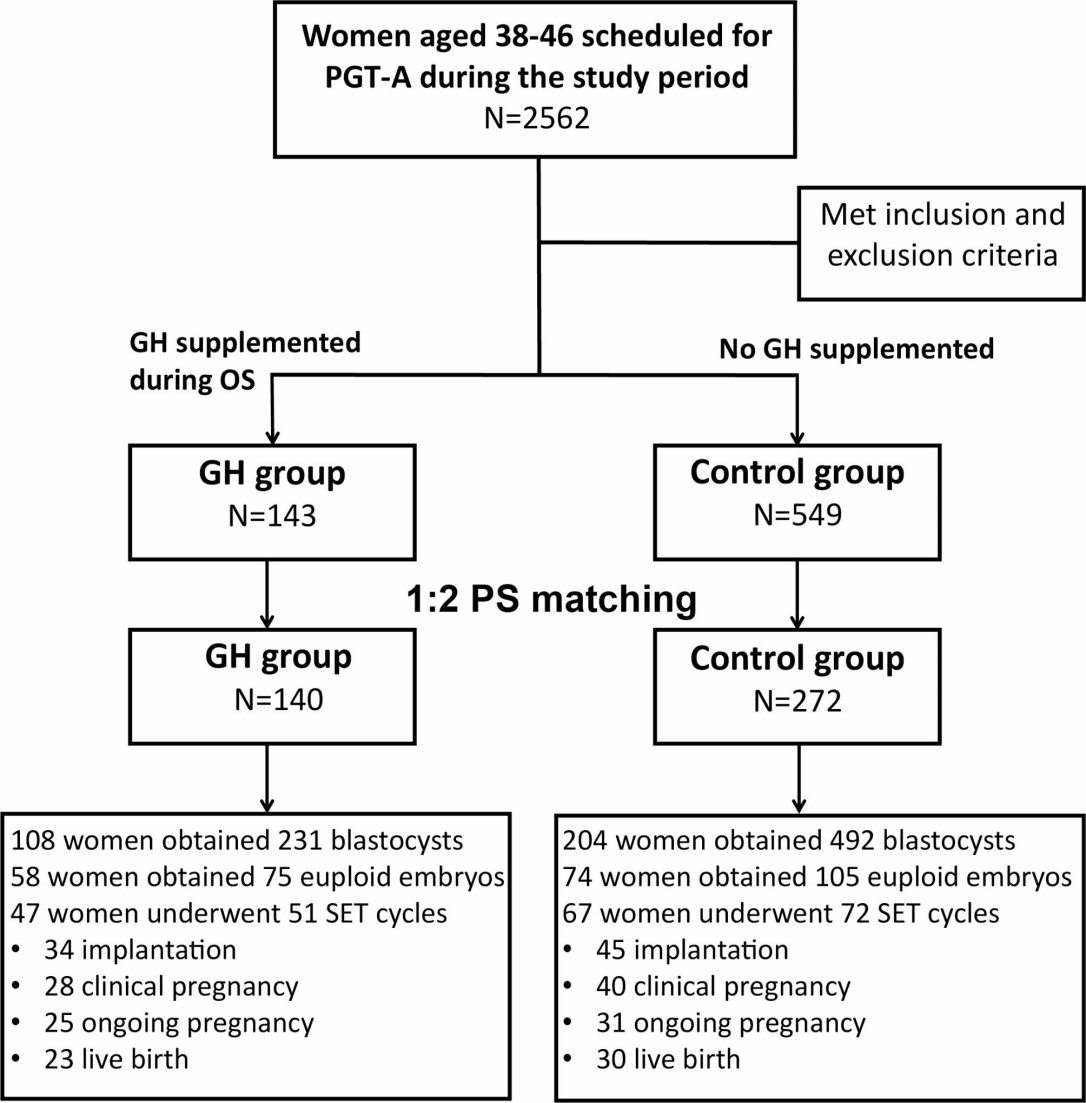
Flowchart detailing the distribution of the two study groups. GH = growth hormone; OS = ovarian stimulation; PGT-A = preimplantation genetic screening; PS = propensity score; SET = single embryo transfer

A total of 692 women with AMA undergoing PGT-A were included in this study, including 143 in the GH group and 549 in the control group (Fig. 1). Before PSM, the number of previous OS cycles in the GH group was significantly higher than that in the control group (P < 0.05), whereas the other baseline and cycle characteristics were not significantly different (Table 1). After PSM, 140 patients in the GH group were successfully matched to 272 patients in the control group, thereby creating highly comparable cohorts with no statistically significant difference in baseline and stimulation characteristics (Table 1).

**Table 1:** Baseline and cycle characteristics of the GH and control groups before/after propensity score matching

	Before PSM			After PSM		
	GH group N = 143	Control group N = 549	P-value	GH group N = 140	Control group N = 272	P-value
**Baseline characteristics**						
Age (years)	41.30 ± 2.31	41.13 ± 2.07	0.38	41.25 ± 2.31	41.20 ± 2.05	0.83
Age distribution, n (%)						
38–40 years	63 (44.06)	243 (44.26)	0.125	63 (45.00)	117 (43.00)	0.38
41–43 years	50 (34.97)	226 (41.17)		49 (35.00)	112 (41.20)	
44–46 years	30 (20.97)	80 (14.57)		28 (20.00)	43 (15.80)	
BMI (kg/m^2^)	21.72 ± 2.41	21.97 ± 2.66	0.31	21.74 ± 2.43	21.68 ± 2.38	0.83
No. of previous OS cycles	3.16 ± 2.00	2.55 ± 1.79	**0.00**	3.06 ± 1.89	2.97 ± 1.99	0.66
Indication for PGT-A, n (%)						
AMA	22 (15.39)	129 (23.50)	0.09	22 (15.70)	45 (16.50)	0.71
AMA with history of PL due to aneuploidy	51 (35.66)	206 (37.52)		51 (36.40)	88 (32.40)	
AMA with RIF	33 (23.08)	94 (17.12)		33 (23.60)	67 (24.60)	
AMA with RPL	37 (25.87)	120 (21.86)		34 (24.30)	72 (26.50)	
Ovarian reserve						
Basal FSH (mIU/mL)	9.99 ± 4.85	10.09 ± 4.90	0.83	9.95 ± 4.85	10.00 ± 4.22	0.92
Basal LH (mIU/mL)	4.75 ± 2.58	4.50 ± 2.30	0.26	4.76 ± 2.60	4.46 ± 2.67	0.22
Basal E2 (pg/ml)	45.87 ± 19.90	42.44 ± 21.94	0.09	45.60 ± 20.00	44.63 ± 23.15	0.67
AMH (ng/mL)	1.54 ± 1.53	1.70 ± 1.41	0.23	1.56 ± 1.54	1.55 ± 1.01	0.93
AFC	8.32 ± 5.33	8.85 ± 6.08	0.35	8.39 ± 5.36	8.41 ± 5.66	0.96
**Cycle characteristics**						
Duration of stimulation (days)	9.36 ± 1.83	9.16 ± 2.75	0.41	9.41 ± 1.80	9.37 ± 2.79	0.87
Total dosage of gonadotropins (ampoules, 75 IU/ampoule)	34.57 ± 12.25	32.09 ± 16.11	0.09	34.66 ± 12.36	34.17 ± 16.94	0.77
Oestradiol on trigger day (pg/ml)	2637.05 ± 1847.10	2844.30 ± 1911.75	0.25	2657.15 ± 1860.78	2779.61 ± 1900.54	0.53
No. of oocytes retrieved	6.97 ± 4.94	7.67 ± 5.51	0.17	7.06 ± 4.95	7.42 ± 5.20	0.50
No. of mature oocytes (MII) retrieved	5.52 ± 3.84	6.19 ± 4.53	0.11	5.59 ± 3.85	6.08 ± 4.45	0.27
Fertilized oocytes (2PN)	4.62 ± 3.60	5.25 ± 3.97	0.09	4.69 ± 3.61	5.21 ± 4.01	0.20
No. of cleavage stage embryos	3.50 ± 2.92	3.97 ± 3.27	0.12	3.54 ± 2.94	3.88 ± 2.36	0.30
No. of high-quality embryos on day 3	2.70 ± 2.58	3.03 ± 2.73	0.20	2.74 ± 2.59	2.96 ± 2.70	0.43
No. of blastocysts	1.64 ± 1.68	1.87 ± 1.89	0.18	1.65 ± 1.69	1.81 ± 1.93	0.40

GH = growth hormone; PSM = propensity score matching; BMI = body mass index; OS = ovarian stimulation; PGT-A = preimplantation genetic screening; AMA = advanced maternal age; PL = pregnancy loss; RIF = repeated implantation failure; RPL = recurrent pregnancy loss; FSH = follicle stimulating hormone; LH = luteinizing hormone; E2 = estradiol; AMH = anti-Mullerian hormone; AFC = antral follicle count; PN = pronuclei.

### PGT-A results between the GH and control groups

There was no significant difference in the biopsy cycle rates between the two groups (Table 2). However, the proportion of cycles which obtained euploid blastocysts were significantly higher in the GH group than that in the control group (41.43% vs. 27.21%, P = 0.00). Blastocysts obtained in the GH group had a significantly higher euploidy rate (32.47% vs. 21.34%, P = 0.00) and significantly lower aneuploidy rate (63.64% vs. 72.56%, P = 0.02) compared to the blastocysts acquired in the control group. The mean euploid blastocyst rate calculated per biopsy cycle (0.35 ± 0.40 vs. 0.21 ± 0.33, P = 0.00) and per OS cycle (0.27 ± 0.38 vs. 0.16 ± 0.30, P = 0.02) were significantly higher in the GH group than those in the control group (Table 2).

**Table 2:** PGT-A cycle outcome of women in the GH and control groups after PSM

	GH group N = 140	Control group N = 272	P-value
No. of OS cycles	140	272	412
No. of OS cycles obtaining no blastocysts, n (%)	32 (22.86)	68 (25.00)	0.63
No. of PGT-A cycles, n (%)	108 (77.14)	204 (75.00)	0.63
No. of cycles obtaining euploid blastocysts n (%, per OS cycle)	58 (41.43)	74 (27.21)	**0.00**
No. of embryos underwent PGT-A, n (%)	231	492	
Euploid blastocysts	75 (32.47)	105 (21.34)	**0.001, 1.772 (1.249–2.514)** ^**a**^
Aneuploid blastocysts	147 (63.64)	357 (72.56)	**0.015, 0.662 (0.474–0.924)** ^**a**^
Mosaic blastocysts	9 (3.90)	30 (6.10)	0.222, 0.624 (0.291–1.338) ^a^
No. of euploid blastocysts acquired			
Per OS cycle	0.54 ± 0.74	0.39 ± 0.75	0.17
Per PGT-A cycle	0.69 ± 0.78	0.51 ± 0.82	0.06
No. of aneuploid blastocysts acquired			
Per OS cycle	1.06 ± 1.38	1.31 ± 1.50	**0.04**
Per PGT-A cycle	1.36 ± 1.43	1.75 ± 1.45	**0.03**
Blastocyst euploid rate			
Per OS cycle	0.27 ± 0.38	0.16 ± 0.30	**0.00**
Per PGT-A cycle	0.35 ± 0.40	0.21 ± 0.33	**0.00**

a. Pearson’s chi-square test was used to compare the between group difference of the number of the denoted row and the sum of the other two rows. The results were presented as p-value, odds ratio (95% confidence intervals). GH = growth hormone; OS = ovarian stimulation; PGT-A = preimplantation genetic screening; PSM = propensity score matching

Subgroup analyses indicated that for patients aged 38–40 years, the proportion of cycles with euploid blastocysts (66.67% vs. 43.59%, P = 0.00), the euploid rate of blastocysts per embryo biopsied (50.58.47% vs. 29.01%, P = 0.02), the mean blastocyst euploid rate calculated per OS cycle (0.40 ± 0.37 vs. 0.24 ± 0.33, P = 0.01) and per biopsy cycle (0.46 ± 0.37 vs. 0.31 ± 0.35, P = 0.01) were significantly higher in the GH group than those in the control group. However, the benefit of GH on these outcome parameters was not significant in patients aged 41–43 years and 44–46 years (Table 3).

**Table 3:** Subgroup analyses of PGT-A outcomes between the GH and control groups after PSM

	GH Group N = 140	Control Group N = 272	P-value	OR(95%CI)
The proportion of cycles which obtained euploid blastocysts*				
Age 38–40 years (n = 180)	42/63 (66.67)	51/117 (43.59)	0.003	2.588 (1.367–4.902)
Age 41–43 years (n = 161)	12/49 (24.49)	19/112 (16.96)	0.265	1.587 (0.701–3.593)
Age 44–46 years (n = 71)	4/28 (14.29)	4/43 (9.3)	0.516	1.625 (0.371–7.112)
The euploid rate of blastocyst per embryo biopsied				
Age 38–40 years (n = 400)	56/138 (40.58)	76/262 (29.01)	0.019	1.671 (1.085–2.575)
Age 41–43 years (n = 236)	15/64 (23.44)	25/172 (14.53)	0.105	1.800 (0.879–2.575)
Age 44–46 years (n = 87)	4/29 (13.79)	4/58 (6.90)	0.250	2.160 (0.499–9.345)
The aneuploid rate of blastocyst per embryo biopsied				
Age 38–40 years (n = 400)	73/138 (52.90)	157/262 (59.92)	0.177	0.751 (0.496–1.138)
Age 41–43 years (n = 236)	49/64 (76.56)	145/172 (84.30)	0.167	0.608 (0.299–1.236)
Age 44–46 years (n = 87)	25/29 (86.21)	54/58 (93.10)	0.250	0.463 (0.107–2.003)
The mosaic rate of blastocyst per embryo biopsied				
Age 38–40 years (n = 400)	9/138 (6.52)	29/262 (11.07)	0.140	0.561 (0.257–1.221)
Age 41–43 years (n = 236)	0/64 (0)	1/172 (0.58)	0.729	/
Age 44–46 years (n = 87)	0/29 (0)	0/58	/	/
Blastocyst euploid rate per OS cycle				
Age 38–40 years (n = 180)	0.40 ± 0.37	0.24 ± 0.33	0.005	
Age 41–43 years (n = 161)	0.19 ± 0.37	0.10 ± 0.27	0.085	
Age 44–46 years (n = 71)	0.13 ± 0.32	0.07 ± 0.23	0.406	
Blastocyst euploid rate per biopsy cycle				
Age 38–40 years (n = 146)	0.46 ± 0.37	0.31 ± 0.35	0.012	
Age 41–43 years (n = 117)	0.26 ± 0.41	0.14 ± 0.30	0.081	
Age 44–46 years (n = 49)	0.19 ± 0.39	0.10 ± 0.27	0.307	

* Data are presented as number of cycles with obtained euploid blastocysts/number of OS cycles, (%)

GH = growth hormone; OR = odds ratio; CI = confidence interval

Multivariate logistic regression analyses showed that GH supplementation was an independent factor in improving the acquisition of euploid embryos in patients with AMA aged 38–46 years (adjusted risk ratio [aRR] = 2.273, P = 0.001). Stratified analyses indicated the significant benefit ofGH supplementation was more pronounced in women aged 38–40 years (aRR = 2.653, P = 0.006). However, there was no association between GH supplementation and the acquisition of euploid embryos in patients aged41–43 years and 44–46 years (Table 4).

**Table 4:** Logistic regression analyses of variables associated with the acquisition of euploid embryos*

Variables	P-value	Adjusted OR	95%CI
**Women aged 38–46 years**			
GH supplement	**0.001**	2.273	1.381–3.745
Age	**0.000**	0.637	0.561–0.723
AFC	**0.000**	1.113	1.064–1.164
**Subgroup- Women aged 38–40 years**			
GH supplement	**0.006**	2.653	1.318–5.348
AFC	**0.000**	1.194	1.106–1.288
**Subgroup-Women aged 41–43 years**			
Age	**0.032**	0.591	0.365–0.956
AMH	**0.022**	1.587	1.068–2.360

*Confounders including GH supplement, age, AMH, AFC, BMI, number of previous OS cycles, duration of stimulation, total dosage of gonadotropins, and estradiol on trigger day, were evaluated using multivariate logistic regression models (backward LR). Covariates were retained in the final adjusted model if they were significantly associated with the outcome parameters (P < 0.05). For the subgroup of women aged 44–46 years, none of the aforementioned variables were significantly associated with the acquisition of euploid embryos; therefore, they are not presented in the table. GH = growth hormone; OR = odds ratio; CI = Confidence interval; AFC = antral follicle count; BMI = body mass index; AMH = anti-Mullerian hormone; OS = ovarian stimulation

### Clinical outcomes after embryo transfer

By the end of the study, 47 patients in the GH group and 67 patients in the control group had undergone embryo transfer cycles, all of which were single euploid blastocyst transfers. Implantation, clinical pregnancy, ongoing pregnancy and live birth outcomes were similar between the GH and control groups (Table 5).

**Table 5:** Clinical outcomes of embryo transfer

	GH group N = 47	Control group N = 67	P-value
No. of embryos transferred	51	72	
Implantation, n (%)	34 (66.67)	45 (62.50)	NS
Clinical pregnancy, n (%)	28 (54.90)	40 (55.56)	NS
Ongoing pregnancy, n (%)	25 (49.02)	31 (43.06)	NS
Live birth, n(%)	23 (45.10)	30 (41.67)	NS
Gestational age at delivery (weeks)	38.82 ± 1.08	38.60 ± 1.12	NS
Birth weight (g)	3260.20 ± 413.62	3275.33 ± 446.25	NS

GH = growth hormone; NS = not significant

## Discussion

In this retrospective cohort study with PSM, GH supplementation was associated with higher proportion of cycles with euploid blastocysts, euploidy blastocyst rate per cohort, and mean euploid blastocyst rate per cycle in women with AMA. Moreover, GH supplementation was an independent factor in improving the acquisition of euploid blastocysts in this population, and the effect was age-dependent, with greater benefits in patients aged 38–40 years. However, for patients aged ≥ 41 years, GH supplementation had no significant effect on increasing the acquisition of euploid embryos during OS. Euploid blastocysts obtained with or without GH supplementation did not differ significantly in clinical outcomes after embryo transfer.

To the best of our knowledge, this is the first study to investigate the effect of GH in IVF from the perspective of the ploidy status of embryos in patients with AMA. Currently, only one study has evaluated the effect of GH supplementation during PGT-A cycles in 41 patients without a priori suspicion of poor outcomes based on their clinical parameters [20]. The research team presented preliminary evidence that GH supplementation in these women is associated with significantly more euploid embryos for transfer. However, their results did not show an increased euploidy rate after GH supplementation, which was explained by the self-controlled design of the study, as a woman’s euploidy rate was inherently predetermined based on her age, genetics, and other factors that would remain the same across cycles within a short study period. Therefore, the authors suggested that the increase in the number of euploid embryos was likely due to more mature oocytes retrieved and, thus, more blastocysts available for biopsy [20]. In the present study, there were no significant differences in the number of oocytes retrieved and blastocysts for biopsy in patients with or without GH supplementation. This may be due to the PSM method that selected patients who were most similar in baseline and stimulation characteristics between the two groups. However, we identified an increased proportion of cycles that obtained ≥ 1 euploid blastocyst for transfer in the GH group, and the blastocyst euploid rate per cohort and per cycle were also higher in patients with AMA who had received GH supplementation. A recent study by Lin et al. revealed that GH-treated mouse oocytes have a significantly lower proportion of morphologically abnormal spindles than control oocytes during in vitro maturation [22]; thus, we hypothesized that GH supplementation might reduce meiotic errors and the occurrence of aneuploidy during oocyte development, thereby improving the proportion of euploid oocytes and reducing the occurrence of aneuploid blastocysts.

As the aneuploidy rate of oocytes increases with age, we stratified the patients by age to investigate whether the effect of GH treatment was age dependent. We found that GH supplementation benefitted the acquisition of euploid embryos in women aged 38–40 years, but the effect was not significant in patients aged ≥ 41 years. These results are consistent with those of a previous study by Keane et al. [21], who reported that women younger than 39 years were more likely to achieve clinical pregnancy with GH supplementation; however, GH did not change the chance of pregnancy for those aged 40 years and older. A study by Skillern et al. [20] showed that GH supplementation significantly increased the number of biopsied and euploid blastocysts in younger patients aged ≤ 37 years, while the benefits were not significant in patients aged > 38 years, which we suppose should be due to the small sample size of only 20 in this subgroup. Thus, we proposed that the positive effect of GH in OS is dependent on age; it was more effective in younger patients, while the benefit was unclear in women of ultra-advanced age.

We compared the implantation, clinical pregnancy, ongoing pregnancy and live birth rates of euploid embryos obtained with or without GH supplementation, and the results showed no differences. Meanwhile, the gestational age at delivery and birth weight of newborns were similar between the two groups (Table 5). These results indicated that GH supplementation during OS did not affect the subsequent development potential of euploid embryos, and therefore further proved that the observed increase in clinical pregnancy, ongoing pregnancy, and live births in existing studies [23–27] should be related to a higher proportion of euploid embryos in the GH group.

There were no significant differences between the two groups in the proportion of patients with mosaic embryos (9/140, 6.43% vs.18/272, 6.62%) and the rate of mosaic embryos per biopsy (9/231, 3.89% vs. 30/492, 6.10%, Table 2). Thus, we infer that GH supplementation might not affect mitotic errors occurred post-zygotically. In our IVF center, the mosaic embryos with low proportion (20–50%) of abnormal cells are retained for genetic counseling and transfer [19]. There are 4 patients who obtained only low proportion mosaic embryos but no euploid embryos in the OS cycle. However, all of them refused to transfer mosaic embryos, thus we did not have data on the outcome of mosaic embryos in the two study groups.

Our study has some limitations. First, this study was observational and retrospective in nature, which can only provide correlation instead of causation of the benefit of GH on embryo euploidy status. Second, there was a potential selection bias as patients in the two groups were not randomized. Although all the first PGT-A cycles during the study period were reviewed and were 1-to-2 matched using propensity scoring, some potential unknown or unmeasured covariates may have led to incomplete matching. For example, affordability may also have been a confounder since patients were required to pay for GH, and it was not possible to be evaluated in our study. Third, this was a single center study which limited the sample size. Although the dataset was relatively large and sufficient patients were included in the two groups, caution need to be made to interpret the age group analyses due to the reduced case number per subgroup, especially for patients with ultra-advanced ages (≥ 44 years). Patients in this age group seeking IVF do not account for a high proportion of patients in IVF centers but were clinically challenging, with an extremely low chance of live birth [28]. We found an improvement in the proportion of euploid embryos with GH supplementation in patients aged 41–43 and 44–46, but the difference was not statistically significant. Randomized placebo-controlled trials (RCT) with larger sample size are needed, and the effect of GH supplementation in women aged ≥ 41 years requires further study.

## Conclusions

In conclusion, GH supplementation during OS is associated with a significantly increased probability of obtaining euploid blastocysts in women aged 38–40 years, but this benefit is not significant in patients aged 41–46 years. Our results might be helpful for AMA patients undergoing PGT-A cycles to obtain a better outcome and meanwhile to avoid over-treatment. Future RCTs are needed to confirm our results and the effect of GH in women aged ≥ 41 years requires further investigation.

## Data Availability

The datasets used and analyzed during the current study are available from the corresponding author on reasonable request. Upon publication, we will share the individual participant data and study protocols that support the findings of this article, provided that they have been deidentified, with no specific end date for availability. These materials will be made available to researchers who submit a sound proposal. Proposals should be directed to xiaoxi_sun@aliyun.com.

## Abbreviations

AFC: Antral follicle count
AMA: Advanced maternal age
AMH: Anti-Mullerian hormone
FSH: Follicular-stimulating hormone
GH: Growth hormone
GHR: Growth hormone receptor
IGF: Insulin-like growth factor
IVF: In vitro fertilization
LH: Luteinizing hormone
PGT-A: Preimplantation genetic testing for aneuploidy
PSM: Propensity score matching
OS: Ovarian stimulation
rh-GH: Recombinant human growth hormone
BMI: Body mass index
RCT: Randomized placebo-controlled trials
